# Reading hominin life history in fossil bones and teeth: methods to test hypotheses regarding its evolution

**DOI:** 10.1002/brv.70132

**Published:** 2026-01-21

**Authors:** Paola Cerrito, Judith M. Burkart, Carel van Schaik

**Affiliations:** ^1^ Department of Evolutionary Anthropology University of Zurich Winterthurerstrasse 190 Zurich 8057 Switzerland; ^2^ Center for the Interdisciplinary Study of Language Evolution (ISLE) University of Zurich Affolternstrasse 56 Zurich 8050 Switzerland; ^3^ Comparative Socioecology Group Max Planck Institute of Animal Behavior Universitätsstrasse 10 Constance 78457 Germany

**Keywords:** life history, cooperative breeding, recording structures, human evolution, dental histology

## Abstract

Human life history is derived compared to that of our closest living relatives, the great apes. It has been suggested that these derived traits are causally related to aspects of our ecology, social behaviour and cognitive abilities. However, resolving this requires that we know the evolutionary trajectory of our distinctive pattern of growth, development, and reproduction. Here, we (*i*) outline these derived features and the theories that have been proposed for their evolution; (*ii*) highlight the major gaps in our knowledge related to adult life history (reproduction and post‐reproductive lifespan) with a review of our current knowledge, which is mainly based on information extracted from fossil teeth and bones; and (*iii*) provide an overview of novel analytical methods that leverage the biology of these hard tissues, to generate new information regarding the evolution of some of our peculiar life‐history traits, such as short interbirth intervals (high reproductive frequency) and a prolonged female post‐reproductive lifespan. Our review of tissue biology and analytical methods focuses on two tissues that are formed continuously during the entire lifespan of the individual and can therefore act as recording structures of adult life: dental cementum and lamellar bone. We conclude by providing specific guidelines for future research to help resolve the following long‐standing question in human evolution: how and when did we switch from independent breeding to cooperative breeding, with its high reproductive frequency? Answering this question is crucial for understanding the evolutionary interplay between reproductive physiology and cooperation as well as for understanding how reproductive division of labour might shape societal structure.

## I. INTRODUCTION

Humans are part of the great ape clade and are the only surviving species of the genus *Homo*. Despite some intra‐specific variation, our (*Homo sapiens*) life‐history profile is significantly different from that of our closest living relatives (chimpanzees *Pan troglodytes* and bonobos *Pan paniscus*). Because these latter species show clear similarities with the other extant great apes (Knott, 2001), it is likely that our life history is derived relative to that of the last common ancestor (LCA) with the *Pan* species.

How, when, and in response to which selective pressures our unique life‐history profile evolved is a major open question in evolutionary anthropology (Hawkes & Paine, 2006; Isler & van Schaik, 2012). Current information on the life history of extinct hominins is mainly limited to ages of dental development (Dean, 2006; Kelley & Bolter, 2013; Robson & Wood, 2008). More information would help us test the various hypotheses regarding the drivers of our life‐history pattern and help us infer the ecological or social conditions that are linked to these changes. For instance, knowing when infants began to be weaned earlier than expected (for a primate our body size) would likely inform us about the presence of allomaternal care, since provisioning of solid food by allomothers generally increases birth rates (Cerrito & Spear, 2022; Isler & van Schaik, 2012).

The aim of this paper is to review the tools available for coaxing life‐history information from the available fossils. Here, we first catalogue the derived features and briefly examine the theoretical interpretation of these changes. After that, we inventory the techniques available to extract life‐history information from fossils, review what has been achieved and explore what can be achieved in future work.

### (1) How are humans different?

Over the decades, extensive data have been collected on mobile hunter–gatherer populations (reviewed in Volk, 2023), allowing us to have a better understanding of what our species' life‐history profile is under the conditions (such as diets, population densities, mobility and mortality patterns) that more closely resemble those pre‐dating the advent and spread of agriculture and modern medical care (Mace, 2000; Robson, van Schaik & Hawkes, 2006). Fundamentally, five major differences are apparent.

**Fig. 1:**
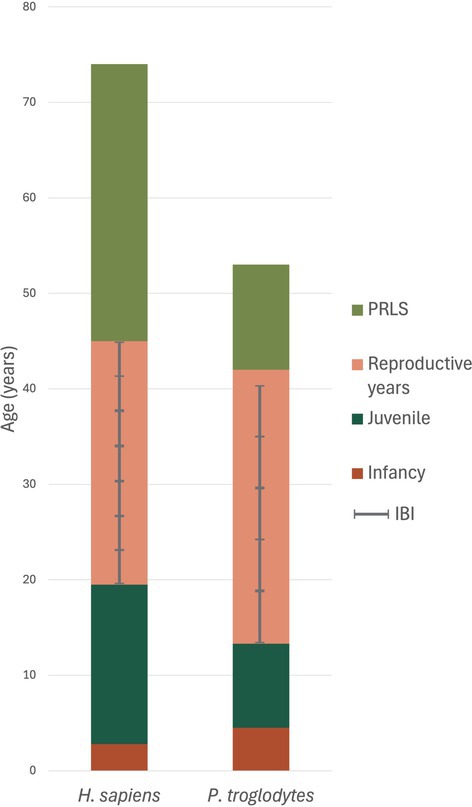
Stacked bar plot representing the duration (in years) of female life‐history stages in *Homo sapiens* and *Pan troglodytes*. Data are from (Robson *et al*., 2006), who compiled data from several primary sources of different populations of hunter–gatherers (for humans). Chimpanzee data are from wild populations (see Thompson *et al*., 2007). We equate the end of infancy with the age at weaning completion, and the beginning of the post‐reproductive lifespan (PRLS) with the age at last birth. Interbirth intervals (IBIs) are overlaid onto the reproductive years. The shorter IBIs of humans allow for an increase in reproductive frequency.

First, humans show a strikingly extended lifespan (Fig. 1). Hunter–gatherer populations have a modal observed maximum lifespan (depending on population) of 68–79 years (Gurven & Kaplan, 2007), while all other great apes have a maximum lifespan of 50 (bonobos) to 58 years (orangutans *Pongo pygmaeus*) (Robson *et al*., 2006) in nature.

Second, a life‐history trait that is linked to a prolonged adult lifespan but distinctive of our species, and of a few non‐terrestrial mammals (Ellis *et al*., 2018), is the presence of a significant (*sensu* Levitis & Lackey, 2011) post‐reproductive lifespan (PRLS) (but see Wood *et al*., 2023), or what one might call midlife menopause.

Third, we observe a delayed age at first reproduction (AFR), estimated for females rather than males to minimize the effect of variable mating systems, relative to great apes. AFR does not very much, even in agricultural societies, based on historical records (Le Bourg *et al*., 1993; Pettay *et al*., 2005). In the absence of birth control, AFR in nomadic foragers is about 19 years, while that of great apes ranges from 10 in gorillas (*Gorilla gorilla*) to 15 in orangutans (Emery Thompson & Sabbi, 2019).

Fourth, human foragers wean infants much earlier than great apes, despite having slowed‐down development, at about 2.5 years (Konner, 2005; Mace, 2000; Robson *et al*., 2006; Walker *et al*., 2006). Accordingly, interbirth intervals (IBIs) can (but do not have to be) much shorter than in great apes. The mean IBI in mobile human hunter–gatherers is 3.7 years (Konner, 2005; Mace, 2000; Robson *et al*., 2006; Walker *et al*., 2006), with solid food introduced at around 6 months. These values are about 80% higher in chimpanzees and 100% higher in orangutans (Robson *et al*., 2006).

Also linked to earlier weaning is the proposal that childhood is an entirely new and unique ontogenetic phase in humans (Bogin, 1997; Konner, 2011; Leigh, 2001). Across primates, by definition, infants turn into juveniles when weaning is completed, such that nutritional independence coincides with the end of nursing (Pereira & Leigh, 2003). Conversely, human children are weaned significantly before they develop the anatomical traits (e.g. mature dentition, attainment of full motor dexterity) that allows them to become nutritionally independent. Childhood is the phase between the end of infancy (complete weaning, at *c*. 2.5 years) and the beginning of the juvenile stage (nutritional independence). During this period children are still nutritionally dependent on others (often including the mother), but no longer through the mother's milk. Thus, childhood is a byproduct of the temporal lag between weaning and nutritional independence rather than (as sometimes argued, e.g. Bogin, 2003) an adaptive delay in independence due to our unusual development. The end of childhood, at around age 8 (Bogin, 1997), coincides closely with what would be the IBI in humans if there were no allomaternal energy input (e.g. Isler & van Schaik, 2012). We will therefore no longer consider childhood as a separate life‐history characteristic.

Fifth, human babies are larger at birth and fatter (Kuzawa, 1998), despite a gestation length that is only 10 to 30 days longer than in other great apes (Emery Thompson & Sabbi, 2019), suggesting a higher rate of investment during gestation. Nonetheless, they are developmentally more altricial, but this difference is made up rapidly and not considered to be relevant to overall life history (Burkart *et al*., 2025; Cusack, Ranzato & Charvet, 2024; Gómez‐Robles *et al*., 2024).

Overall, relative to the presumed ancestral state, humans live longer and develop more slowly, but females stop giving birth well before senescing yet have larger and more frequent babies while they reproduce.

### (2) Why are humans different?

Across mammals in general, life‐history patterns show a fast–slow gradient where, in a given animal, all phases of life and reproduction run faster or slower (MacArthur & Wilson, 1967), such that increased longevity is usually correlated with later weaning, later AFR, etc. Intriguingly, compared to our closest relatives, our life history is neither overall faster or slower: some aspects are accelerated (i.e. short spacing between births and early weaning) while others are decelerated (i.e. the presence of a prolonged female post‐reproductive lifespan, increased longevity). An increase in adult longevity is generally considered to be the product of lower unavoidable adult mortality (Charnov, 1991, 1993; Stearns, 1976, 1989). This is a straightforward correlate of the difference in the survival curve of human foragers in relation to the other great apes (Gurven & Kaplan, 2007). This difference in turn is thought to reflect a shift in ecology from potential prey status to that of a top predator, and from periodic starvation to buffering through extensive food sharing (Alexander, 1990), both of which reduced mortality relative to the ancestral state. This in turn favoured longer lifespans and the requisite investment during development into the defence and repair mechanisms that enable long lifespans (Chen & Maklakov, 2012).

The slowdown of development is not an inevitable byproduct of greater longevity, since reduced risk of starvation accompanied by higher food availability could, in principle, speed up development (Sibly *et al*., 1985). The best explanation for our slower development is instead our far larger adult brain (Barton & Capellini, 2011; Isler & van Schaik, 2009; Schuppli *et al*., 2016). There is strong evidence that the overall rate of development slows down with brain size, because larger brains develop and mature more slowly (Heldstab *et al*., 2020; Workman *et al*., 2013) and physical growth cannot run far ahead of brain development (Worthman, 1993). Yet, while the growth of brain and body mass is initially isometric (Halley, 2016), it is not clear whether brain growth universally places an absolute limit on the developmental process or merely faces a trade‐off with physical growth (as in humans: Kuzawa *et al*., 2014). In the latter case, higher net energy input could increase overall growth rates. The great similarity in growth rates of humans and chimpanzees (Leigh, 2001) suggests that energy inputs into human children (due to allomaternal inputs) result in slightly higher overall growth rates in humans – a pattern less likely due to an absolute limit on brain development than to competition between brain and body for limiting resources during growth. Competition also explains the adolescent growth spurt, which, although hard to detect because of variation in its timing, is present in most large‐brained species (Leigh, 1996), but quite pronounced in humans (Bogin, 2003). Indeed, it has been shown that there is a trade‐off between brain growth (primarily during childhood) and body growth, for which we see a spurt in adolescence (Kuzawa *et al*., 2014).

Midlife menopause, which represents a dramatic decoupling between somatic and reproductive senescence, remains difficult to explain in a convincing way (Kirkwood & Shanley, 2010). It is intuitive that somatic and reproductive senescence are linked: an organism does not benefit from expending energy in maintaining a functional germline if age‐related bodily decline prevents the successful rearing of offspring (Jones *et al*., 2014). As a matter of fact, most vertebrates show a coordinated somatic and reproductive decline. Nevertheless, in a small number of species, the two are decoupled, meaning that either somatic senescence is delayed or reproductive senescence is accelerated. Either way, the resulting trait is an extended PRLS, which evolved independently at least three times in mammals (Ellis *et al*., 2018): once in primates (in humans) and twice in cetaceans. Based on recent research on chimpanzees (Wood *et al*., 2023), the most likely scenario for the evolution of a significant PRLS in humans is the delay of somatic senescence, while maintaining a constant rate of female reproductive decline (Hawkes *et al*., 1998; Kim, McQueen & Hawkes, 2019). By virtue of the small comparative sample, phylogenetically informed comparisons on the evolution and adaptive significance of this trait are difficult. Hence, various hypotheses continue to coexist unresolved. Thus, some maintain that a PRLS is an adaptive trait present in several species as a result of selection (Brent *et al*., 2015; Cant & Johnstone, 2008; Hawkes *et al*., 1998; Lahdenperä *et al*., 2004), while others regard it as unique to humans but consistent with life‐history allometries (Judge & Carey, 2000) and therefore not necessarily the result of selection; some others argue it is phylogenetically very widespread (Austad, 1997; Cohen, 2004; Tully & Lambert, 2011). The driving force in humans, however, remains contested: it can be seen as an adaptive response to continuing foraging efficiency but declining reproductive rate with age (Hawkes *et al*., 1998), or as ‘the best of a bad job’ due to rapidly rising birth defects and perinatal mortality of both mother and child in natural‐fertility populations without sophisticated medical care (van Schaik, 2016). While the hypotheses of selection for menopause are controversial, the consequences of midlife menopause are profound: first, it creates a source of significant allomaternal care (Page *et al*., 2019, 2025), with a possible impact on birth rates (see next paragraph), and second it changes the operational sex ratio in the local population, with potentially important social consequences, in particular the strengthening of mate guarding and thus pair bonds (Nitschke *et al*., 2025).

The higher birth rates of women may be due to various factors. First, humans have higher basal or resting metabolic rates than great apes, which permits a higher energy turnover (Pontzer *et al*., 2016) and therefore higher birth rates. However, comparative studies do not find much evidence for a link between metabolic turnover and reproductive rate [Harvey, Pagel & Rees, 1991; Trevelyan, Harvey & Pagel, 1990; but see Auer *et al*. (2018) for ectotherms]. Second, unlike great ape females, human females are provisioned by others, especially males and grandmothers (Kramer, 2010). Cooperative breeders, in which females receive energetic support in the form of provisioning, infant carrying or infant huddling, have higher birth rates (Cerrito & Spear, 2022; Isler & van Schaik, 2012). Finally, allonursing, by optimizing the utility of the effect of milk on growth and development, may improve the overall efficiency. Allonursing is absent among great apes but near‐universal among human foragers (Hewlett & Winn, 2014) and known to increase reproductive output per unit time across mammals (Cerrito & Spear, 2022). Allomaternal provisioning and allonursing may be complementary.

This brief excursion indicates that knowledge of the timing of the various changes in life‐history features during human evolution would provide interesting information about the roles of ecological dominance, brain size, metabolic rate, allomaternal care, food sharing and allonursing, which would significantly improve our understanding of the major transitions during our evolution.

## II. HOMININ LIFE HISTORY

So far, efforts to understand human life history have mostly been based on comparisons among extant species, by inferring the value of those variables that have been shown, empirically, to correlate with them (e.g. body mass, dental eruption times). However, recent methodological advances may enable us to reconstruct directly the changes that occurred during human evolution, and therefore to understand which traits co‐emerged with each other, why and when. We therefore follow Robson & Wood (2008) in discriminating between two types of variables which are often conflated: life‐history variables (LHVs) and life‐history‐related variables (LHRVs); and proceed to review our knowledge of LHVs in extinct hominins (Table 1).

**Table 1:** Availability of life history variables (LHVs) and life‐history‐related variables (LHRVs) for fossil hominin taxa.

Variable	Type	Available for extinct taxa
Gestation length	LHV	No
Age at first reproduction	LHV	Not yet
Age at weaning	LHV	Yes – but only for some hominin taxa
Interbirth intervals	LHV	Not yet – but is related to age at weaning
Mean lifespan (longevity)	LHV	No – but yes for Upper Paleolithic *H. sapiens*
Age at last reproduction	LHV	Not yet
Maximum lifespan	LHV	Not yet
Adult body mass	LHRV	Yes
Adult brain mass	LHRV	Yes
Neonatal body mass	LHRV	Yes
Dental formation times	LHRV	Yes
Dental eruption times	LHRV	Yes

Modified from Robson & Wood (2008). Variables indicated as ‘not yet’ are those for which we here propose methods to obtain them.

### (1) Longevity

The accuracy in estimating the chronological age of hard tissues (bones and teeth) decreases drastically after growth is completed. This is because most skeletal ageing methods are based on the evaluation of the degree of formation of a structure (e.g. dental development and obliteration of cranial sutures; Martrille *et al*., 2007), for which the pace of temporal progression is known in modern populations. Aging in adults is based on the extent of degenerative processes (e.g. dental wear), which are more idiosyncratic than developmental ones (McFadden, Cave & Oxenham, 2019). There is evidence that the pace of development of extinct hominins was faster than ours (Bromage & Dean, 1985; Dean *et al*., 2001) with enamel formation rates of both australopiths and early *Homo* closer to those of apes than modern humans. Therefore, anatomical developmental milestones can indicate a certain ontogenetic phase but not the chronological age at which it is reached. Current hypotheses regarding the evolution of longevity during hominization are therefore predicated upon the assumption of collinearity between body or brain mass and maximum lifespan (Robson & Wood, 2008; Schwartz, 2012).

Estimates based on regressions of maximum lifespan on body mass in extant primates (Judge & Carey, 2000) suggest a major increase in longevity between *Homo habilis* (52–56 years) and *H. erectus* (60–63 years) occurring roughly 1.7–2 million years ago (Lieberman *et al*., 2021; Tobias, 2006), concurrently with a significant acceleration in brain size increase (Gómez‐Robles *et al*., 2017). There is reason to be cautious in accepting values inferred from regressions based on other species, however, since what we observe in extant humans is exactly the departure from such regressions. Another approach to estimating longevity is based on dental wear. Caspari & Lee (2004) estimated the proportion of individuals within a given age class (albeit with only two age classes: young and old adults) in four broadly defined hominin groups (Australopithecines, Early *Homo*, Neanderthals and Early Upper Paleolithic humans). They concluded that old age became frequent only late [from *c*. 50 thousand years ago (kya)] in human evolution, among the Upper Paleolithic *H. sapiens*, corroborating previous results on Neanderthal mortality patterns (Trinkaus, 1995). Obviously, this approach assumes that dental wear rates remain approximately constant across time, despite different diets and food‐processing techniques.

Another recent approach uses ancient DNA and is based on the analysis of the density of CpG islands on the genome, which are target sites for DNA methylation and have been shown to correlate (*r* ^2^ = 0.76) with longevity (Mayne *et al*., 2019). Results based on this method concluded that Neanderthal and Denisovan maximum lifespans were about 37.8 years (Mayne *et al*., 2019), although there is some room for doubt since these values are well below those for earlier hominins or even extant great apes.

### (2) Significant post‐reproductive lifespan

Currently, there is very little evidence on this topic, limited essentially to the evidence of older‐aged people first appearing in Upper Paleolithic *H. sapiens* (Caspari & Lee, 2004). Since the age at menopause in chimpanzees is approximately that of modern humans (Wood *et al*., 2023), it is parsimonious to assume that it was also similar in our LCA. Hence, our significant PRLS is arguably a consequence of our prolonged lifespan. However, for this very plausible hypothesis to be tested, actual data are necessary, not only on longevity but also on age at menopause. Currently, the latter is not available for any fossil hominin.

### (3) Ontogeny and age at first reproduction

The bulk of our current knowledge regarding hominin life history relies on patterns of growth and development. There are mainly two types of studies: those based on known correlations between dental development and the attainment of ontogenetic milestones, which therefore provide *relative* data; and those based on histological methods, which can produce absolute chronological values (reviewed in Dean, 2006).

Several correlative studies between molar emergence and life‐history variables have been carried out since seminal work by Schultz (1949). Because they are based on extant humans and great apes (Jeanson *et al*., 2016; Smith, 1984, 1989; Smith, Crummett & Brandt, 1994), the regressions they generated are not necessarily applicable to extinct hominins. Indeed, recent virtual histological work (which allows visualization and assignment of a chronological age to ontogenetically incremental structures in teeth, but see Section IV.1 for methodological details) has shown that the dental ontogeny of early hominins (*Paranthropus* and *Australopithecus*) (Smith *et al*., 2015), early *Homo* (Smith *et al*., 2015), *H. erectus* (Dean, 2016; Zollikofer *et al*., 2024), and Neanderthals (Smith *et al*., 2010*b*) varied and was not simply intermediate between extant apes and modern humans.

With the advent of histological approaches to dental anthropology, it has become possible to assign an *absolute* chronology to dental ontogeny in individual fossil specimens. This approach reduces the uncertainty from inferences based on the comparative approach. Since the seminal work by Bromage & Dean (1985) studies on hominin growth and development have increased exponentially. We now know that the pace of maturation in *Paranthropus* (Dean *et al*., 2020), early *Homo* and *H. erectus* was faster than our own (Dean *et al*., 2001; Dean & Smith, 2009; Zollikofer *et al*., 2024). Work on ontogenetic patterns of cranial morphology (Antón & Leigh, 2003) has suggested that *H. erectus* lacked a clear indication of an adolescent growth spurt, which is characteristic of *H. sapiens*. Nonetheless, the high degree of morphological variation within *H. erectus* has been argued, despite the large geographic and temporal spread, to be indicative of a developmental plasticity unmatched even by Neanderthals but still inferior to our own (Antón *et al*., 2016).

The bulk of recent work on ontogenetic patterns has focused on Neanderthals (Dean, Stringer & Bromage, 1986; Macchiarelli *et al*., 2006; Mahoney *et al*., 2021; McGrath *et al*., 2021; Nava *et al*., 2020; Rozzi & De Castro, 2004; Smith *et al*., 2007, 2010*b*, 2018). Together, these data suggest that Neanderthals' ontogenetic trajectories were accelerated compared to our own. Indeed, the earliest evidence of modern human life history is found in an early (*c*. 300 kya) *H. sapiens* specimen (Smith *et al*., 2007) from Jebel Irhoud (Morocco), which is also the locality where the earliest members of our species have been found (Hublin *et al*., 2017). We therefore do not find evidence for modern human‐like growth patterns in any extinct hominin species, although modelling approaches have attempted to infer this information for mid‐Pleistocene *Homo* (Durand, Ipina & de Castro, 2000).

However, despite all these advances, almost nothing is known regarding the LHV related to developmental pace that is clearly derived in modern humans: AFR. With the exception of one study (Cerrito *et al*., 2022*b*) that leveraged virtual [synchrotron X‐ray micro‐computed tomography (micro‐CT)] histology of dental cementum to estimate AFR in the Krapina Neanderthals (15.5 and 16.6 years for two distinct individuals), we still lack this information for all other hominins.

### (4) Age at weaning

While weaning is a notoriously ambiguous term (van Noordwijk *et al*., 2013*a*), here we use it to indicate the first departure from a 100% milk diet, as recovered from fossil teeth. The earliest hominin species for which there is evidence regarding age at weaning is *Australopithecus africanus*. Recent research suggests that, at least in one individual, a predominantly maternal‐milk diet ceased at about 1 year of age (Joannes‐Boyau *et al*., 2019), after which cyclical elemental patterns are observed in the enamel, likely indicating irregular food availability. This work supports the long‐standing assumption that during hominin evolution there was a trend towards a reduction in the age at weaning, which is earlier in our species compared to extant *Pan* species. Indeed, while there is intra‐specific variation, a marked increase in solid food intake occurs at about 1 year of age in wild chimpanzee populations (Lonsdorf *et al*., 2020) and between 1 and 1.5 years in orangutans (van Noordwijk *et al*., 2013*b*).

There is direct evidence for age at weaning in Neanderthals as well. Estimates for the onset of intake of solid foods, based on different individuals, vary between *c*. 9 months (Smith *et al*., 2018), *c*. 7 months (Austin *et al*., 2013) and *c*. 6 months (Nava *et al*., 2020). In modern humans, the modal age at initial introduction of solid foods is *c*. 6 months (Sellen, 2001). Collectively, while there was certainly within‐species variability, the current research suggests that the beginning of solid food introduction in Neanderthals occurred at a similar age as in recent humans. Even if we accept that their growth pattern was slower than ours (Smith *et al*., 2010*b*), it appears that a human‐like age at weaning had evolved either before the modern human–Neanderthal divergence, or evolved independently in the two lineages, which seems to be the less‐parsimonious option. This suggests that either both had significant allomaternal input or that easily digestible and processed food was prepared by the mother herself. Knowledge of interbirth intervals would provide information on whether, and how soon, the mother was already occupied with nursing another offspring thus limiting her ability to care for the previous one.

### (5) Interbirth intervals

Age at weaning, as measured by the departure from a 100% milk diet, is only a poor proxy for interbirth intervals. There is a long time interval between the age of first food intake and age at full weaning (cessation of all suckling), and reproduction may only start again after suckling stops. Hence, to gain knowledge about the evolution of female reproductive frequency, age at weaning is not sufficient and actual data on the timing of parturition events must be derived from fossil specimens. So far, some methods have been developed (reviewed in Section IV.2 and Table 3), which among extinct hominins have only been applied to Neanderthals (Cerrito *et al*., 2022*b*).

### (6) Summary

In summary, some elements of recent human life history suggest a ‘live slow’ strategy, while others suggest a ‘live fast’ schedule. The broad evolutionary questions underlying life‐history research are whether our modern life‐history package developed as a single module, or rather in a mosaic fashion, and when the various shifts in scheduling appeared. Shortened IBIs and prolonged PRLSs are the most notably derived features of our life‐history strategy (Robson *et al*., 2006). Yet, we do not know whether they are correlated in their emergence during hominin evolution. Humans are the only primates that exhibit a marked decoupling between age at weaning and emergence of the first permanent molar (Robson *et al*., 2006). Some have suggested the possibility of a prolonged infant dependency on adult food supplementation linked to some form of cooperative breeding (Hawkes *et al*., 1998; Hrdy, 2011; Kramer, 2010; Kramer & Otárola‐Castillo, 2015; van Schaik & Burkart, 2010). To assess the need for cooperative breeding and its possible relation to grandmothering (Hawkes *et al*., 1998), as opposed to other forms of alloparenting, such as those seen in common marmosets (*Callithrix jacchus*) where help is provided by fathers and older siblings (Brügger & Burkart, 2021), it is important to know a female's rate of giving birth as well as PRLS.

In the next section, we review the analytical methods that may enable researchers to infer these key features (birth rate and age at menopause) to move towards a better understanding of these outstanding questions in human evolution.

## III. THE BIOLOGY OF RECORDING STRUCTURES

Most of what we know regarding the timing of LHVs in fossil specimens derives from the histomorphological or elemental analysis of teeth. These function as recording structures (Klevezal, 1995) which in nature encompass animal tissues as diverse as shells, scales, otoliths, bones, enamel, dentine, cementum, claws, nails, horns and even ear plugs (Trumble *et al*., 2018). All recording structures are periodically layered, and because these layers are sequentially deposited, they are commonly referred to as ‘growth layers’. Like tree rings, each layer is the visible result of changing physiological states of the organism. Each tissue presents specific differences in the morphology of its layers. For example, in cementum and dentine more‐ and less‐transparent bands are visible, while in horn, ridges alternate with grooves. These features are the constituting elements of each recording structure, and determine the existence of the periodically layered appearance.

Recording structures can be categorized according to three temporal parameters (Klevezal, 1995): (*i*) sensitivity, (*ii*) period of registration, and (*iii*) persistence of the record (Table 2). The sensitivity of a structure is a function of its growth rate. The period of registration relates to the temporal range during which a given structure forms new layers. In humans, for example, enamel layers stop forming when the tooth cap is completed, while new layers of bone are deposited throughout the life of the individual. The persistence of a record is a function of the turnover rate of a tissue. For example, enamel does not turnover, while periosteal bone is resorbed and remodelled in response to both mechanical and physiological demands. This remodelling makes the persistence of the record highly variable, even within different bones of the same individual (Fahy *et al*., 2017). In such instances, the record ceases to exist once the portion of tissue in which it was recorded is resorbed.

**Table 2:** The three fundamental parameters of the four different ‘recording structures’ present in primates (modified from Cerrito *et al*., 2022*b*).

Structure	Sensitivity	Growth rate	Period of registration	Persistence of a record
Lamellar Bone	Multidien	*c*. 9 μm/*c*. 7 days	8th week after conception to end of life	Several years, but variable depending on bone, population and individual
Enamel	Circadian and multidien	2.5–4.5 μm/day	Tooth initiation to tooth crown completion	Lifetime
Dentine	Circadian and multidien	3–8 μm/day	Tooth initiation to tooth completion	Lifetime
Cementum	Annual	1–3 μm/year	Tooth gingival emergence to death	Lifetime

These parameters determine the suitability of each structure for the different questions in anthropological research. Growth rate determines the spatial resolution that the analytical instrument of choice (optical microscopy, electron microscopy, micro‐computed tomography, etc.) should have, in order to detect the desired events. The growth rate values indicated are for extant *H. sapiens*. Multidien periodicity varies across primates, it is on average 7 days for humans, but for example only 4 days in rhesus macaques (*Macaca mulatta*). A full overview of multidien periodicities in primates and extinct hominins can be found in (Bromage *et al*., 2009).

The recording structures present in primates and potentially retained in fossils are bone, dentine, cementum, and enamel. Table 2 summarizes the temporal parameters of each of these four structures. Given the focus of this article on adult life‐history events, chiefly IBIs and age at last reproduction, we briefly outline the biology of the only two recording structures that continue secretion into adulthood: dental cementum and bone.

### (1) Cementum

**Fig. 2:**
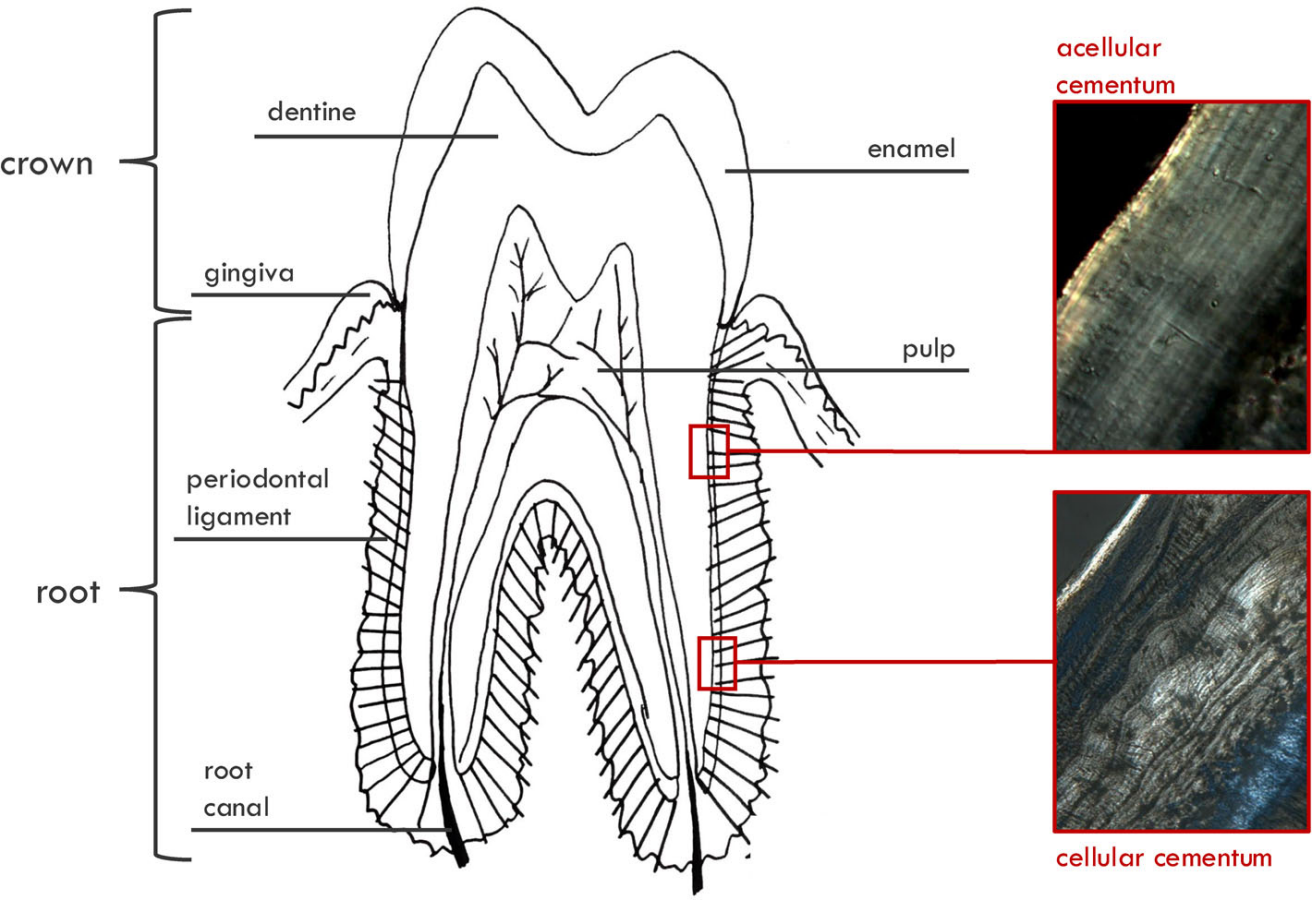
The histological composition of a tooth. Diagram of longitudinal section; two micrographs of human acellular (top) and cellular cementum (bottom). Modified from Dean *et al*. (2018).

Teeth are constituted by an enamel cap covering the coronal aspect of the dentine and an underlying dentine core, which contains a pulp chamber. Cementum covers the tooth root (Fig. 2), has the function of attaching and protecting the tooth within the dental alveoli of the jaws (Schroeder, 2012), and is not subject to remodelling throughout life.

Apical cementum serves mostly a compensatory function against tooth movement, has a rapid secretion rate and contains cementocytes that remain embedded during formation, giving rise to cellular cementum. Incremental bands are visible in apical (cellular) cementum, but also in cervical (acellular) cementum. The two are very different, but the more regularly layered cervical acellular cementum is considered the region of choice for the analysis of both physiologically impactful events and age‐at‐death estimation. Traditionally, the alternating light and dark cementum bands that provide its characteristic layered appearance are thought to be determined by different mineralization levels. Dark bands (in transmitted light microscopy) are thought to correspond to slower forming cementum, while lighter bands correspond to more rapidly forming cementum, and therefore it is the very accentuated dark bands that are thought to be indicative of periods of physiological stress. However, a debate exists regarding whether light or dark bands are more mineralized (Colard *et al*., 2016; Lieberman, 1994) and also whether the layered appearance is determined by differing degrees of mineralization or by changes in the mineral and collagen fibre orientation (Cool *et al*., 2002).

### (2) Bone

Bone, like cementum, contains an organic and an inorganic component. Depending on the way that collagen fibre bundles, osteocytes (bone‐forming cells), and vascular canals are organized, bone can vary widely in both structure and histology. Bone can be woven or lamellar. Lamellar bone is highly organized and consists of parallel layers of lamellae laid down appositionally. Primary lamellar bone can originate endosteally (the inner surface of the bone, lining the medullary cavity) or periosteally (the outer surface), while secondary lamellar bone is formed later in life and results from remodelling of primary bone. Lamellar bone conserves a layered appearance and can be considered as a recording structure of physiologically significant events (Cerrito *et al*., 2022*a*).

## IV. LEVERAGING DENTAL AND OSTEOLOGICAL DYNAMIC FEATURES TO RECONSTRUCT LIFE‐HISTORY EVOLUTION

To contextualize the use of skeletal remains (which encompass teeth and bones) to infer life‐history events, we review the current knowledge regarding the systemic relationships between skeletal tissues and both inner (Gross, 1998) and outer environment. The participation of the skeleton to whole‐organism physiology is achieved primarily *via* the modelling and remodelling processes of bone. Osteoblasts, differentiating into osteocytes and bone‐lining cells, deposit the organic matrix and modulate mineralization. Osteoclasts, by contrast, dissolve and break down bone. *Via* some simple chemical processes and associated cellular specializations, bone contributes not only to the regulation of calcium homeostasis but also to the physiology of reproduction, and to fat and energy metabolism, in addition to responding to obvious mechanical functions.

Hence, in extant mammals, skeletal physiology is inextricably linked to energy metabolism, endocrine regulation and global mineral homeostasis (DiGirolamo, Clemens & Kousteni, 2012). As such, it reflects and participates in both inner metabolic rhythms (Bromage *et al*., 2016) and environmental cyclicity (Doherty, Ghalambor & Donahue, 2015). Likewise, it responds and contributes to a variety of physiologically impactful events (Dirks *et al*., 2002; Lemmers *et al*., 2021) and also to environmental changes (Bromage *et al*., 2011; Cipriano, 2002; Hamilton, Fernandez & Nelson, 2021).

An organism's metabolism encompasses all those chemical processes implicated in the conversion of food into energy (e.g. adenosine triphosphate) and structural molecules (e.g. proteins and nucleic acids) and in the elimination of the waste generated by these conversions. Metabolism modulates the pace of life (energy/matter converted per unit time) according to three main periodicities (Table 2): (*i*) circadian, which is extremely conserved phylogenetically; (*ii*) yearly, which is clearly mediated by the environment; and (*iii*) multidien, which is instead correlated with body mass, although with a phylogenetic contribution. These three periodicities regulate the appearance of layered structures in hard tissues (Table 2) (Boyde & Bromage, 2021), therefore providing the basis for both age‐at‐death estimation and for the chronological reconstruction of the physiologically impactful events that manifest themselves as slight changes in the morphology of these periodic increments.

Having a grasp of each tissue's periodicity, recording timeline and of the complex physiology regulating its formation and remodelling is important to understand which questions can be addressed by analysing a given structure, and which cannot. First, the circadian rhythm is manifested in the daily cross striations of enamel, which result from tissue matrix secretion being regulated by circadian transcription factors present in the enamel‐forming cells, ameloblasts (Lacruz *et al*., 2012).

Second, a much longer periodicity than the daily one also modulates metabolism. It is related to seasonal changes in both sunlight (Gorman, de Courten & Lucas, 2019) and temperature (Seebacher, 2009). Vitamin D is the principal mediator between sunlight exposure (specifically, ultraviolet‐B photons) and hard tissue metabolism. All skeletal‐forming cells, including cementoblasts, are endowed with Vitamin D receptors (VDRs) (Bikle, 2012). VDRs have both a rapid action, by opening calcium ion (Ca^2+^) channels and stimulating Ca^2+^ absorption, and a slower one involved in regulating skeleton‐related gene transcription, which is therefore modulated seasonally (Darling *et al*., 2014) and reflected in yearly growth lines (Foster & Hujoel, 2018). The link between temperature and skeletal metabolism is less direct, mediated by the relationship between osteoblasts and energetic homeostasis. Specifically, osteoblasts (and likely cementoblasts given their extreme similarity, but the literature is scarce) regulate insulin production and adipose tissue metabolism thus integrating their high energetic requirements with the overall energy balance of the body (Dirckx *et al*., 2019), which is in turn affected by thermoregulatory demands (Seebacher, 2009).

Finally, the multidien periodicity, known as the Havers–Halberg Oscillation (HHO) (Bromage *et al*., 2009), regulates aspects of metabolic activity that in turn contribute to regulating the enormous variation observed in the pace of life. Research has revealed that this periodicity is associated with body mass, which in turn is linked with tissue‐specific metabolic rates. These rates are higher in small mammals than in large ones (MacAvoy, Arneson & Bassett, 2006), and correspondingly the frequency of HHO periodicity of smaller animals is higher than that of larger ones (Bromage *et al*., 2016). Since this rhythm is linked to cell proliferation rates, it also affects transcription regulation, protein synthesis and Ca^2+^ concentrations (Thompson, 2011). HHO periodicity also regulates lamellar increments, with one lamella corresponding to one Retzius periodicity (RP). In dentine and enamel, the number of daily cross striations between two RPs can be used to derive the value of the RP interval (or HHO periodicity) since the RP value is constant throughout an individual's life [but see Mahoney *et al*. (2017) for a discussion on how HHO may vary within an individual during childhood]. The characterization of HHO values for many extinct hominin species (Hogg *et al*., 2020; Modesto‐Mata *et al*., 2020), has thus allowed inferences regarding their general ‘pace of life’, but not specific estimates of ages at life‐history milestones.

### (1) Age estimation

Since the enamel of deciduous teeth begins formation during prenatal life, there is an RP increment corresponding to birth (an event which is physiologically impactful for the newborn), which is histologically accentuated (Weber & Eisenmann, 1971) and elementally distinct (reviewed in Nava *et al*., 2024). This so‐called neonatal line provides a starting point (birth) from which all further (and previous) increments can be assigned a chronological value. Because of the staggered fashion in which different teeth develop, it is possible to match lines in earlier‐forming teeth to those in later‐forming ones, and to build chronologies that span the formation time of multiple teeth (e.g. Zollikofer *et al*., 2024). This methodology, which is based on the presence of the circadian and multidien periodicities, has been used to estimate the age at death of immature hominin fossil remains (Bromage & Dean, 1985; Lacruz, Rozzi & Bromage, 2005; Smith *et al*., 2010*b*).

Age at death of adult individuals can be estimated based on the presence of a life‐long yearly periodicity in the deposition of cementum. This methodology is less precise than the previous one based on enamel, given the lack of a neonatal line as well as assumptions that must be made regarding the age at which cementoblasts begin secretion (which roughly corresponds to the gingival emergence of the tooth). Prior knowledge of dental formation times is therefore necessary to proceed with age‐at‐death estimation based on the count of yearly cementum annuli. These estimates are available for many extant primate species (AlQahtani, Hector & Liversidge, 2010; Bolter & Zihlman, 2011; Kralick *et al*., 2017; Liversidge, 2008; Smith, 2016; Smith *et al*., 2010*a*; Trotter, Hixon & MacDonald, 1977; Zihlman, Bolter & Boesch, 2004) but are more complicated to devise for extinct species. Nonetheless, cementum annulation (Wittwer‐Backofen, 2012) has been used to estimate age at death in a wide range of mammals, including rhesus macaques *Macaca mulatta* (Kay, Rasmussen & Beard, 1984; Kay & Cant, 1988), horses *Equus ferus caballus* (Prilepskaya *et al*., 2020), several cervid species, polar bears *Ursus maritimus* (Christensen‐Dalsgaard *et al*., 2010), bats (Cool, Bennet & Romaniuk, 1994) extinct stem‐mammals (Newham *et al*., 2020), extinct hominins (Cerrito *et al*., 2022*b*; van Heteren *et al*., 2023) and both contemporary (Sultana *et al*., 2021; Wittwer‐Backofen, Gampe & Vaupel, 2004) and archaeological humans (Huffman & Antoine, 2010; Le Cabec *et al*., 2019; Tanner *et al*., 2021).

### (2) Menopause, age at first reproduction, age at weaning, interbirth intervals

Both elemental and histological changes in bone and cementum have been detected in association with a variety of life‐history‐related events such as reproduction (Carrel, 1994; Cerrito *et al*., 2020, 2021, 2022*a*, 2023; Medill *et al*., 2010; Von Biela *et al*., 2008; Wittwer‐Backofen *et al*., 2004) and menopause (Cerrito *et al*., 2020, 2022*b*). The methods to infer the four relevant variables (menopause, age at first birth, age at weaning, interbirth intervals) are extremely similar, while the recording structure in which to detect them (Table 2) changes. A brief review of the way in which the skeleton participates in whole‐organism physiology, including reproductive physiology, can be helpful to understand which questions can be asked, and why.

During physiologically impactful events (such as birth, weaning, menopause, etc.) an organism must adapt in order to maintain homeostasis (Gross, 1998), while optimally balancing energetic availability. Because of the complex and systemic nature of organisms, the homeostatic response involves several different systems and organs, including the skeleton. It is therefore not surprising that changes in reproductive physiology and in nutrition correlate with skeletal changes that can be observed *via* the histological and elemental analysis of teeth and bones.

Being the largest store of both calcium and phosphate, bone has a significant role in regulating organismal homeostasis. During gestation, parathyroid hormone (PTH) and vitamin D increase serum concentrations of phosphate and calcium by both resorbing bone tissue and increasing intestinal absorption. Conversely, these concentrations are decreased *via* the signalling activity of bone‐derived fibroblast growth factor 23 (FGF23) (Bergwitz & Jüppner, 2010). Bone is also implicated in regulating feeding and energy balance *via* a hypothalamic–osteoblastic–endocrine loop in which leptin suppresses bone formation and increases bone resorption by delivering signals to osteoclasts (Ducy *et al*., 2000). The reciprocal to this loop is the one initiated by osteocalcin, a bone‐ and cementum‐specific protein expressed in osteocytes, osteoblasts and cementocytes (Kagayama *et al*., 1997), that increases insulin levels (Lee *et al*., 2007) and stimulates testosterone biosynthesis (Karsenty & Oury, 2014). Hence, as reproductive physiology impacts both an organism's relative energy allocation and its absolute energetic requirements, bone resorption and deposition are correlated with gestation and lactation; these same events affect the matrix secretion rate of cementum, giving rise to more highly mineralized annuli in correspondence with moments of high energetic throughput.

Cementum (Elzay, 1964) and bone (Cauley, 2015; Gillies, 2017; Khosla, Oursler & Monroe, 2012) are also highly responsive to circulating oestrogen levels, which promote calcium storage in anticipation of reproductive events. Conversely, decreases in oestrogen, such as those associated with menopause, are linked with increased osteoclastic activity and consequent net bone tissue loss (Ahlborg *et al*., 2003) across females of diverse ethnicities (Finkelstein *et al*., 2008). Decreasing oestrogen levels are also correlated with changes in size and orientation of hydroxyapatite (HA) crystals (Turunen *et al*., 2016) as well as with altered cortical bone porosity and mineralization (Sharma *et al*., 2018).

Other hormones associated with reproduction, prolactin (PRL) and oxytocin (OT), also have documented effects on the skeleton. PRL receptors are expressed by osteoblasts, and PRL receptor knock‐out mice show decreased bone mass (Clément‐Lacroix *et al*., 1999) resulting from increased bone turnover with net bone loss (Seriwatanachai *et al*., 2008). OT regulates both bone (Colaianni *et al*., 2012) and cementum (Ge *et al*., 2019) by increasing the proliferation, migration and differentiation of cells, leading to a net increase in tissue. This is consistent with the reproductive function of OT and with the large amounts of calcium and phosphorus that are transferred, *via* lactation, from the mother to the developing infant (Kovacs, 2016), thus resulting in the need to recoup bone mass rapidly after lactation.

In the young, the changes in diet associated with weaning determine corresponding changes in the elemental composition of the developing dentition, such that the transition from a maternal milk diet to either formula or solid foods has been detected and timed in walruses (*Odobenus rosmarus divergens*) (Clark, Horstmann & Misarti, 2020*a*), humans (Li *et al*., 2022) and macaques (Austin *et al*., 2013; Cerrito *et al*., 2021, 2023).

Similarly, since in many populations dietary intake changes seasonally or geographically, *via* the elemental analysis of hard tissues, it is also possible to detect seasonality (Carvalho *et al*., 2001; Cerrito *et al*., 2023; Klevezal & Shishlina, 2001; Pryor *et al*., 2020; Wedel, 2007) and mobility patterns in mammals (Borić & Price, 2013; Copeland *et al*., 2011; Lugli *et al*., 2019).

Changes in zinc concentrations in cementum have been reported to reflect the onset of sexual maturity in female walruses (Clark, Horstmann & Misarti, 2020*b*), whereas declining fertility has been shown to be correlated with increased copper concentrations in the teeth of female rats (Rahnama, 2002). Furthermore, lead and cobalt concentrations in human dentine are sexually dimorphic (Kumagai *et al*., 2012), as are the relative concentrations of major and minor elements in macaque cementum (Cerrito *et al*., 2023).

Several studies have detected histological changes in cementum associated with female reproductive events across a number of mammalian species, including black bears (*Ursus americanus*) (Coy & Garshelis, 1992), sea otters (*Enhydra lutris kenyoni*) (Von Biela *et al*., 2008), humans (Cerrito *et al*., 2020, 2022*b*) and macaques (Cerrito *et al*., 2021). Similarly, accentuated lines in dentine are associated with parturitions in humans (Dean & Elamin, 2014).

Finally, a few studies have reported the possibility of recovering hormonal concentrations embedded in the dental tissues of humans (Nejad *et al*., 2016; Quade, Chazot & Gowland, 2021; Quade *et al*., 2023; Wu *et al*., 2024), extant marine mammals (Hudson, Matthews & Watt, 2021) and extinct terrestrial mammals (Cherney *et al*., 2023). Although the approach by Hudson *et al*. (2021) does not yet have the spatial resolution necessary to detect changes over time, it is particularly promising for human life‐history research, because it captures differing concentrations of progesterone and testosterone for individuals of different age classes and reproductive phases. It could likely be profitably applied to investigate whether a female has already undergone menopause or not.

A summary of the recording tissue and analysis of choice for each variable is reported in Table 3. Unfortunately, most of the methods reported in Table 3 are destructive. With the exception of age at death in adults and infants, which can be inferred *via* histological analysis alone and can therefore be carried out non‐destructively using synchrotron X‐ray micro‐CT, the identification of a specific life‐history event requires elemental analysis, which (at least for now) requires exposing the internal structure of the tooth and therefore sectioning it. Leveraging taphonomically broken teeth can potentially allow the characterization of the internal elemental composition, and therefore the detection and timing of life‐history events. However, future work is necessary to validate this approach.

**Table 3:** Summary of the life‐history variables that can be recovered from hard tissues, together with the type of tissue, methodology and some relevant references.

Life‐history variable	Tissue	Method	References
Age at weaning	Enamel (deciduous teeth)	Histological + elemental analysis	Nava *et al*. (2024); Smith *et al*. (2023)
Age at menarche	Cementum (permanent teeth)	Histological analysis*	Cerrito *et al*. (2022*b*)
Reproductive events in females & birth rates	Dentine (permanent 3rd molar)	Histological analysis*, ^#^	Dean & Elamin (2014)
Reproductive events in females & birth rates	Cementum (permanent molars)	Histological + Elemental analysis*	Cerrito *et al*. (2020, 2023); Wittwer‐Backofen *et al*. (2004)
Menopause	Cementum (permanent molars)	Histological analysis*	Cerrito *et al*. (2020, 2022*b*); Hudson *et al*. (2021)
Reproductive status (fertile/post‐fertile)	Primary lamellar bone	Histological + elemental analysis^$^	Cerrito *et al*. (2022*a*)
Reproductive status (fertile/post‐fertile)	Bulk bone and cementum	Steroid analysis^@^	Cherney *et al*. (2023); Wu *et al*. (2024)
Infant age at death	Enamel (deciduous teeth)	Histological analysis	Bromage & Dean (1985)
Adult age at death	Cementum (permanent teeth)	Histological analysis	Le Cabec *et al*. (2019); Newham *et al*. (2021); Wittwer‐Backofen *et al*. (2004)

* Additional method development is necessary to determine the elemental signature of the event in order to differentiate its histological signal from that of other physiologically impactful events.

^#^ Only captures reproductive events occurring until dentine formation is completed (*c*. 19 years of age).

^$^ Further research needed to validate this on *H. sapiens*.

^@^ Further research needed to test methods for oestrogen in bone and cementum.

In sum, while fossilized teeth and bones are the only biological remnants of our extinct ancestors, it is nonetheless possible to recover in them traces of our diet, mobility, and life history. Hopefully, as efforts continue to be made to improve the methods at our disposal to interrogate the fossil record, we can gain an ever‐more cohesive and integrative understanding of the many changes that occurred to achieve our current life‐history profile.

## V. CONCLUSIONS

(1) Human life history is derived compared to that of our closest living relatives, with some aspects being accelerated (earlier weaning, shorter interbirth intervals) and others being decelerated (later age at first birth, increased longevity). It is still unknown when and how, in response to which selective pressures, and in association with which social changes, our peculiar life‐history pattern evolved.
(2) Traditionally, inferences about the life‐history scheduling of extinct hominins have been based on regressions on extant taxa between life‐history‐related variables (such as brain and body mass, or dental development timing) and life‐history traits. However, there is reason to be cautious in accepting values inferred from regressions since what we observe in extant humans is exactly the departure from such regressions. Hence, research on hominin life‐history evolution aims to detect when and how our lineage started deviating from the relationships between life‐history‐related variables and life‐history traits observed in other apes.
(3) It is therefore essential to develop methods capable of inferring direct chronological data from skeletal remains (bones and teeth) – the constituents of the hominin fossil record. Dental and osteological microstructures serve as a reliable archive of an individual's life‐history milestones, such as weaning, reproduction and cessation of fertility. These skeletal indicators collectively provide a robust framework for assessing the evolution of our species' life history.
(4) The application of high‐resolution imaging and elemental analysis of hominin dental enamel (which ceases formation during childhood) has greatly enhanced our understanding of hominin early life and weaning patterns. These same methods can be modified and applied to two skeletal tissues that continue formation throughout life (bone and dental cementum), allowing us to recover adult life‐history events.
(5) Challenges remain in accurately reconstructing life‐history traits from fossilized remains. The main challenge is the one regarding the destructiveness of the analytical techniques. While histology can be carried out non‐destructively *via* synchrotron X‐ray micro‐CT, histological analysis is merely indicative of a physiologically altering event. Yet, to understand the cause of the event (e.g. illness *versus* reproduction) elemental analysis is necessary. For now, despite the development of protocols that greatly optimize the process, this type of analysis could only be done in destructive ways. Future work should aim at collecting elemental data from naturally exposed dental roots (e.g. from taphonomically broken teeth).
(6) A very promising future line of research is aimed at recovering the hormonal concentrations embedded within teeth. Recent work has proved successful at recovering cortisol concentrations in dental enamel and steroid hormones in dentine. The expansion of this methodology to include other skeletal tissues and other hormones could prove invaluable.
(7) In sum, the study of hominin life history through hard tissue analysis remains a crucial aspect of paleoanthropology. By refining methodologies and broadening interdisciplinary collaborations, it is becoming possible to test broad and far‐reaching hypotheses (e.g. the ‘grandmother hypothesis’; the ‘cooperative breeding hypothesis’) regarding our peculiar life‐history strategy and its associated social (e.g. reproductive division of labour) and cognitive (e.g. prosociality) traits.
